# Oculomotor Dance Task: Implications for Audio–Visual-Cued Spatial Learning

**DOI:** 10.3390/vision10030039

**Published:** 2026-07-01

**Authors:** Michael Petrovski, Salwa Beheiry, Udichi U. Das, Simran Rooprai, Ashkan Karimi, Royze J. Simon, Rachel J. Bar, Sintayehu Wami, Joseph F. X. DeSouza

**Affiliations:** 1 Center for Vision Research, Department of Psychology, York University, 4700 Keele Street, Toronto, ON M3J 1P3, Canada; michael.petrovski00@gmail.com (M.P.); salwabeheiry.psych@gmail.com (S.B.); udichiudas@gmail.com (U.U.D.); ashkan70@yorku.ca (A.K.); royze25@my.yorku.ca (R.J.S.); wamisd@yorku.ca (S.W.); 2 Interdisciplinary Graduate Studies, York University, 4700 Keele Street, Toronto, ON M3J 1P3, Canada; srooprai@yorku.ca; 3 Canada’s National Ballet School, 400 Jarvis St, Toronto, ON M4Y 2G6, Canada; rbar@nbs-enb.ca; 4 Connected Minds: Neural and Machine Systems for a Healthy, Just Society, York University, 4700 Keele Street, Toronto, ON M3J 1P3, Canada; 5 Vision: Science to Application (VISTA), Multisensory Neuroscience Translational Laboratory, York University, 4700 Keele Street, Toronto, ON M3J 1P3, Canada

**Keywords:** motor learning, eye-tracking, neuroplasticity, dancing

## Abstract

This study aims to address whether a new visual–motor-based learning paradigm with music can potentially promote neuroplasticity and create new interventional tools, building upon prior research that shows behavioral and putative neural changes following dance-based neurorehabilitation in people with Parkinson’s disease (PD). Eye movements of 10 healthy adult participants, aged 20–25, were tracked using the Eyelink 1000 Plus system during a 68 s eye dance sequence. The experiment consisted of a learning phase, where participants observed the sequence five times with 30 s breaks, and a performance phase, where they performed the sequence five times from memory on a gray screen without visual cues. Results showed a significant improvement in percentage of total movements performed correctly between the first session (g1; M = 40%, SD = 7.2%) and the last session (g5; M = 69.7%, SD = 22.8%). Similarly, there was significant improvement in the average time on beat from the intended choreography timing, between the first session (g1; M = 0.29, SD = 0.06) and the fifth session (g5; M = 0.46, SD = 0.12). These findings suggest that eye movement choreography has the potential to be learned within healthy adults.

## 1. Introduction

Motor sequence learning (MSL) involves the coordination of both oculomotor and manual motor systems through the practiced repetition of a fixed sequence of actions, resulting in automatized execution of movement [1]. While extensive research regarding MSL has been explored involving neural bases of behavior [2,3], the oculomotor context remains an emerging field of MSL. Typical design of MSL tasks generally assess reaction times using limb movement in response to stimuli [1,4,5,6,7]. Although part of a smaller subset of the MSL literature, various studies have found that eye movements predict MSL similar to that of physical movement [8,9,10], and have activation patterns closely resembling physical motor response [1,6,10]. Other studies have found auditory stimuli to enhance MSL [11], aiding response to visual stimuli. The use of auditory cues synchronously within sequence learning has been associated with increased development of explicit knowledge of learned sequences [12]. These combined cues also effectively reduce latency of oculomotor tasks [13]. In addition, cues of this nature were found to aid in movement sequences that occur over longer periods of time [11,14]. Understanding the components behind sequence learning, the combination of audio and visual cues reinforce behaviors, showing greater results together rather than individually, and creates a significant demand for task design to seamlessly incorporate both facets.

It is increasingly critical to define the underlying mechanisms of motor sequence learning, namely the neurological communications between aforementioned areas of interest. Neuroplasticity is the brain’s ability to reorganize neural pathways in response to degeneration or loss of function within such pathways [15]. There is some scant evidence that has shown neuroplasticity occurring within the cortical oculomotor system. Neural plasticity within the oculomotor system for the superior colliculus (SC) was observed in monkeys, and neural firing from these SC neurons were more efficient for the same eye movements when repeated [15]. Additionally, in humans, the oculomotor system, during repeated eye exercises over 18 min, improved cognitive aspects of the task in accuracy and recall of RSVP letters compared to non-exercise conditions [16]. The resulting efficiency in neuronal activation can show instances in which oculomotor movements exhibit adaptive changes through repetition and neural plasticity.

To prompt neuroplastic change is to engage brain function of specified regions of interest with a task and to understand the compensations or new connections made. Various methods of quantifying neuroplastic changes have been documented [3,17,18,19,20]; however, the developments brought about by physical intervention or tasks related to movement will be pertinent to the following body of work. Systematic reviews have highlighted the importance of physical exercise or engagement in improving executive function and wellbeing in old age [21,22,23]. Even in tasks involving minimal physical effort, greater activations of regions involved, such as frontal eye fields (FEF) [24], SC [25], cerebellar oculomotor vermis [26], and SMA-related motor networks, have been shown [1,8,10,27]. In addition, it has been noted that eye tracking proves to be a promising tool for assessment and clinical applications, as it can potentially be applied towards measurement of neuroplastic changes in neurodegenerative diseases (NDs) [28,29,30]. Providing analysis of specific areas involved in neuroplastic change brought about through motor engagement, whether ocular or physical, remains pivotal.

As a form of physical movement combined with audio influence, and common amongst all cultures and ages, dance has great quantifiable effect on MSL and neuroplasticity [31,32,33]. Dance actively engages an individual physically, cognitively, and sensorially, and it has been widely recognized as a practical intervention to promote neuroplasticity and execute MSL [17,21,22,34,35,36]. While a vast amount of the literature involving measures of neuroplasticity within the last decade suggests physical exercise or dance can lead to improvements or mitigation of impairments, the need for simplified and accessible tasks is paramount. The physical demands of exercise and other coordinated movement, such as dance, continue to pose the challenge of being inaccessible to various groups. It has been widely urged within the surrounding literature to create simplified versions of dance interventions [22,34] and physical exercise interventions [32]. For old-aged, ND, or individuals with disabilities, the severity of cognitive or physical decline is a key issue [37,38], and it is acknowledged that interventions should come to address individuals beyond the mild–moderate scale.

The aim with the present eye-tracking task is to develop a feasible, non-exertive task that minimizes the physical response needed from a participant whilst still activating similar regions of the brain to that of coordinated physical movement and auditory cues, such as dance. Additionally, the absence of a social component while dancing is a novel aspect to discern how learning through this paradigm may differ. Within the current study, the usage of eye movement as an alternative to physical movement in MSL was investigated. In combination with auditory cues of a musical piece, the additional goal of diversifying the current literature on multisensory sequence learning was sought. We hypothesized that a within-subjects design with five sessions of eye dance practice over 5 weeks of time would result in improvements within sequence-specific learning, namely performance accuracy and timing precision.

## 2. Materials and Methods

### 2.1. Participants

Ten healthy adults were recruited from the student population at York University, all of whom provided written informed consent. Exclusion criteria for the study were visual or auditory impairment. A total of *N* = 10 (female = 8, male = 2; ages: 20–25, M = 22) were all tested at York University.

### 2.2. Apparatus and Stimuli

**Figure 1:**
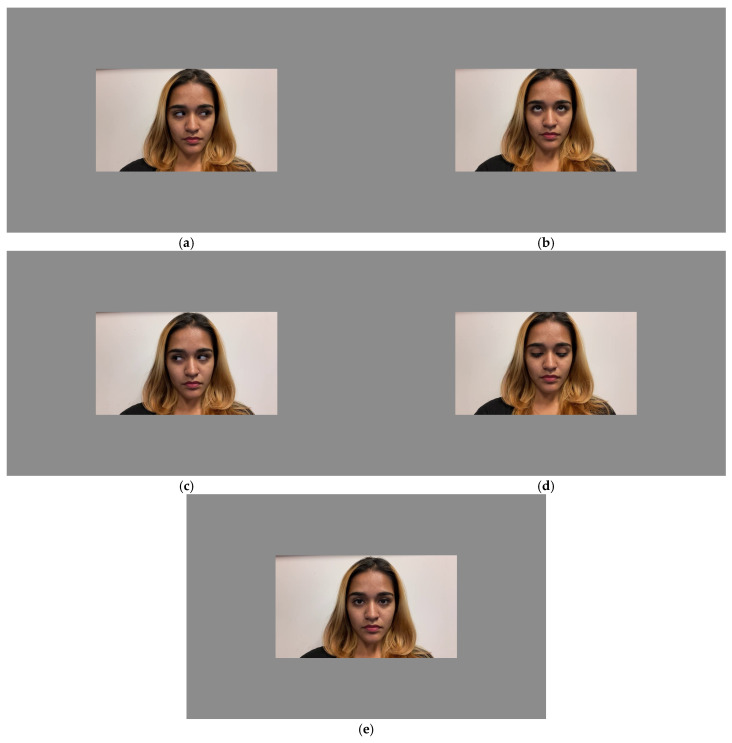
Training interface. Interface was shown to each participant for 68 s with accompanying music and choreography that they were instructed to follow with eye movements. Movements were directionally cued as (**a**) right, (**b**) up, (**c**) left, (**d**) down, and (**e**) center.

**Figure 2:**
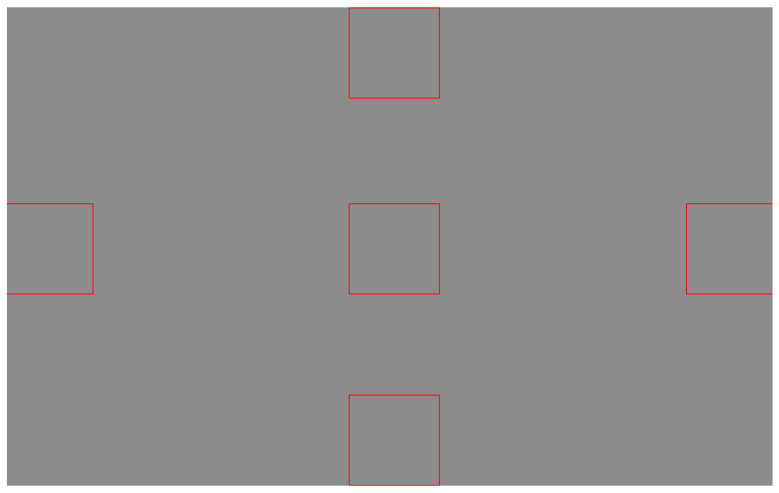
Live scoring interface. Interface was shown to each participant for 68 s with accompanying music as played in training trials. The red boxes are coordinated with the eye movements denoted in Figure 1.

Stimuli were displayed on a built-in LED iMac monitor (Apple Inc., Cupertino, CA, USA; 1680 × 1050 pixels). A headrest was set to ensure stability of participant gaze to the stimuli and clear tracking of the eyes. Viewing distance of the display to headrest was measured to be 55 cm. The headrest was adjusted to ensure gaze was matched to the direct center of screen. Through training trials, a gray background was presented with an 848 × 480 pixel video centered within the display of a woman directing eye movements of the overall dance pattern (Figure 1a–e). The runtime of the video and accompanying audio piece is 68 s. Through performance trials, a gray background was presented with 5 red outlined squares, each measuring 200 × 200 pixels (Figure 2). The squares were located on the display and labeled as the following positions: center (840, 525), up (840, 100), down (840, 950), left (100, 525), and right (1580, 525). All eye movements were directionally cued along the X and Y axes and recorded within an eye-tracking paradigm on an Eyelink 1000 plus (EYELINK I CL v5.04 Sep 25 2014 CAMERA: Eyelink GL Version 1.2 Sensor = AJ7).

### 2.3. Procedure

Participants were taught a complex eye movement sequence to a 68 s musical piece by watching a choreographed eye dance pattern over 5 sessions. The video and music of the dance were played five times, with thirty-second break intervals featuring a gray interface with no audio in between. The set of prior events is referred to as the learning phase of the test. Following the learning phase, participants are tasked to perform the same sequence of eye movements using only the musical piece and target markers on a gray interface. Executions of the dance sequence were recorded five times, with a thirty-second break and a gray interface similar to the learning phase, and are referred to as the performance phase. At the end of each 68 s performance recording, 5 out of the 30 s within the break consisted of a color flash of the interface corresponding to the accuracy of steps correctly made. Three colors were shown—red, yellow, and green—corresponding to total score being <33% correct, 33% < x < 66% correct, and >66% correct, respectively. The visual reinforcer was shown to cue accuracy of the pattern recorded of each participant. Participants were also asked to rate their performance after each trial on a 1–100% scale. After the learning and performance phases, the session was completed, and participants were instructed to complete their next session within 7–10 days.

### 2.4. Outcome Measures

**Figure 3:**
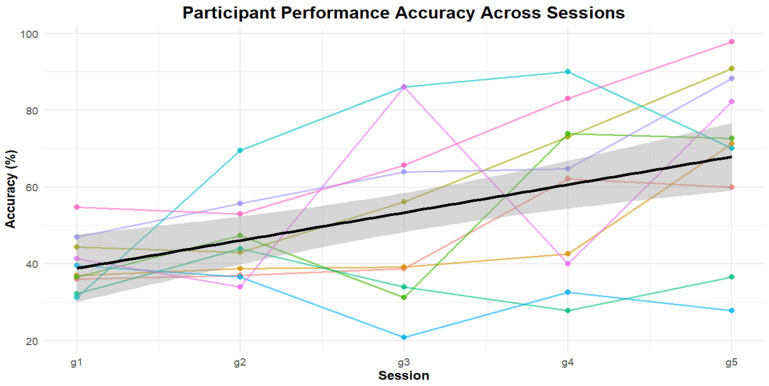
Participant performance accuracy across sessions. Accuracy was recorded based on the proportion of movements correctly executed to the dance sequence (X/46). A line of best fit and shaded 95% confidence interval are shown as well.

**Figure 4:**
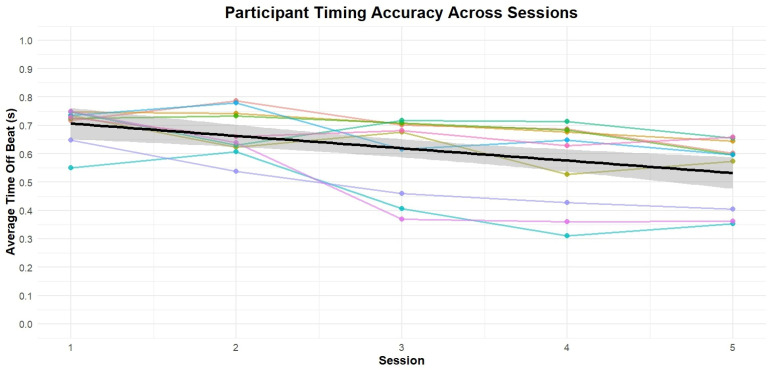
Participant timing precision across sessions. Participant timing was measured based on proximity to the movements synced to video runtime. A line of best fit and shaded 95% confidence interval are shown as well.

Two dependent variables were derived from the performance phase to quantify learning outcomes of the dance. Performance accuracy was defined as the proportion of eye movements executed within spatial thresholds of targets, calculated as the number of correct movements divided by the total number of movements within the instructed choreography, and expressed as a percentage (Figure 3). Timing accuracy was defined as the absolute deviation in seconds between the participant’s eye movement and the corresponding cued movement onset in the stimulus video, with a lower deviation indicating higher precision (Figure 4). Both measures were calculated for each trial and averaged within each session for statistical analysis.

### 2.5. Statistical Analysis

**Figure 5:**
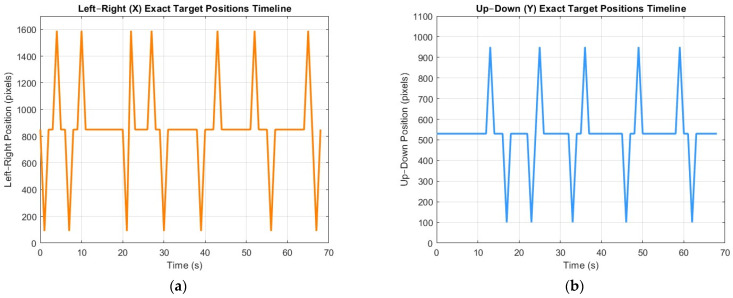
XY value positions of training performance. Exact sequence positions shown in pixels of expected (**a**) X values and (**b**) Y values with video/audio timestamp.

**Figure 6:**
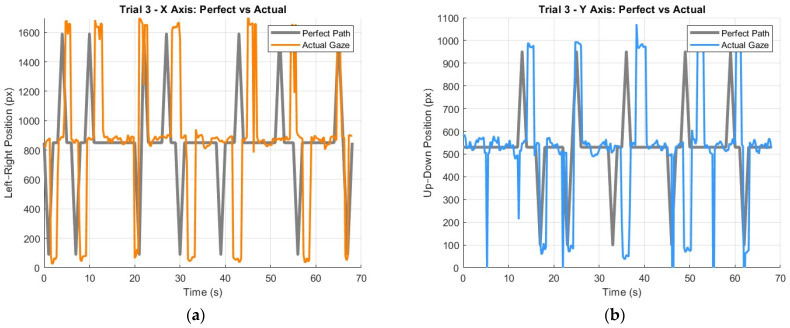
Participant trial position and timing of actual performance. Exact sequence positions shown in pixels of an individual participant’s performance in session 5. (**a**) X values and (**b**) Y values compared from training position to actual performance with video/audio timestamp.

Data plotting and analysis were conducted using software R (version 4.3.1, R Foundation for Statistical Computing, Vienna, Austria) and MATLAB (R2024b, MathWorks Inc., Natnick, MA, USA). Results of accuracy statistics were found using a repeated-measures ANOVA. Eye gaze data was sampled every 2 ms, and all blinks and positions off-screen were omitted from data processing. Target thresholds were located within 200 pixel squares around exact targets (Figure 2). Movements were only marked if 200 samples remained within target threshold (Figure 5a,b), then compared with the dance sequence (Figure 6a,b) to be matched. One-way repeated-measures ANOVAs were selected for statistical analysis of both performance and timing accuracy to assess learning over time, with Mauchly’s test to confirm sphericity and Greenhouse–Geisser as preferred for statistical correction. Both analyses were performed with 95% confidence intervals and a significance threshold at *p* ≤ 0.05.

## 3. Results

### Performance and Timing Accuracy

Repeated-measures ANOVA revealed a significant main effect of sessions on performance accuracy, with F(4, 36) = 6.99, *p* < 0.001, an effect size of η^2^_G_ = 0.26, and Mauchly’s test confirmed that sphericity was met (*p* = 0.32). Additional repeated-measures ANOVA revealed a significant main effect of sessions on timing precision, with F(4, 36) = 11.67, *p* < 0.001, and an effect size of η^2^_G_ = 0.25. Mauchly’s test indicated sphericity was violated (*p* = 0.022); the Greenhouse–Geisser correction confirmed the effect remained significant (*p* = 0.009).

Post hoc pairwise comparisons using Bonferroni correction showed that session 5’s performance accuracy was significantly higher than Session 1 (*p* = 0.008). No other session pairs differed significantly, though the difference in the first and last sessions suggests a gradual increase in performance across each session, leading to significant improvement.

Post hoc pairwise comparisons using Bonferroni correction showed that timing accuracy of movements improved significantly between the early and later sessions, with significant differences observed between sessions 1 and 4 (*p* = 0.044), sessions 1 and 5 (*p* = 0.004), sessions 2 and 4 (*p* = 0.030), sessions 2 and 5 (*p* = 0.007), and sessions 3 and 5 (*p* = 0.007). No significant differences were found between later sessions (g4 and g5, *p* = 1.00), suggesting that timing accuracy plateaued prior to the end of training.

## 4. Discussion

This study investigated the potential of MSL by explicitly using oculomotor function and auditory cues. This novel visual–motor sequence learning task demonstrated significant improvements in both performance accuracy and timing precision, suggesting procedural learning and cognitive–motor coordination can be enhanced through gaze-based repetition. The statistical analyses evidently show a well-defined learning curve, suggesting the efficacy of the task in consolidation of learning following practice. Further analysis of the timing precision can be used to suggest audio cues played a significant role in learning of the sequence. Participants also tended to lag behind audio cue timestamps, in line with findings from Han et al. [13] and Leow et al. [14], which suggests auditory stimuli influence MSL preceding oculomotor movement. Furthermore, it can be suggested that audio cues played congruently with visual stimuli encourages stronger development of explicit knowledge within reaction tasks compared to solely visual tasks [39,40]. The distinct roles of audio and visual stimuli towards influencing eye movement provides a strong foundation for future MSL studies to consist of ND or tetraplegic populations [30], therefore expanding the field on severe motor deficit groups. By using this focused oculomotor approach, the task may potentially aid in designing non-invasive, low-burden rehabilitation tools for enhancing functional outcomes. This provides the opportunity, should interventional and rehabilitative use prove effective, for the task to be simplified for frequent/daily use and delivered using web-based delivery and web-camera recordings.

Various structures of the brain are associated with motor sequence learning and further to this, the associated oculomotor components of learning are expected to be pertinent to the results within the current body of work. To elaborate, while MSL is multifaceted due to various regions of the brain involved, the basal ganglia has been cited throughout the prior literature as a core region associated with learning and reward systems [41,42]. Another region of interest is the ventral tegmental area (VTA), also associated with reward systems [43,44]. These defined areas that encompass the reward system and drive learning mechanisms can be fully traced using fMRI to account for increased functional connectivity by measuring cortical white matter [3,19]. Furthermore, such increases are noted in post-training after MSL tasks [2]; similar results would likely be evident should the present study be replicated with a fMRI component. To further elucidate the mechanisms behind MSL, neurotransmitter functions and measurements must also be accounted for. Once more, neurotransmitter functions of MSL are varied, but namely dopamine [18,45] and GABA [18,20] are promoted by visual tasks [33] and by physical activity [31,46]. As previously elaborated, the oculomotor system of the brain has potential to mimic results of physical activity and prompt neurotransmitter signaling. Areas of the oculomotor circuit to be included in further analyses include the supplementary motor area [2,35,47,48], the frontal eye field [10,47,48], and the supplementary eye field [47], as these are crucial in MSL [24]. This novel eye-tracking task in future studies should therefore incorporate a fMRI scanning element to further explore this oculomotor task for potential results comparable to that of other MSL tasks.

Many eye movements are made while people are engaging in learning dance [14,17,34,35,36,49,50] and are involved in directing their gaze toward the movements of the body, to the teacher, and to other dancers [49]. Interestingly, receiving no visual stimuli while learning a dance does not improve or hinder performance outcomes [51]. It is made clear that similar results from oculomotor influence can benefit those with ND and/or those with physical disabilities. Various forms of exercise or dance have been identified previously within the body of work as promoting putative neuroplastic effects [34]. While limited motor movements of the body would likely pose differing results to typical eye–limb motor sequence tasks, the results found in a solely oculomotor task could expand knowledge of an area that lacks specific motor interventions. PD is an ideal population where this novel oculomotor task can be applied, as they experience deficits in areas directly engaged by this learning paradigm [52]. The use of musical cues and reinforcement feedback may promote dopaminergic engagement related to reward circuitry, which is particularly relevant for the PD population as well [33,50]. While not tested within the present study, the exploration of dopaminergic reinforcement involving the score indicator would be of great interest for future studies. Furthermore, dopaminergic changes can modulate anxiety [53,54], which future applications of the task can aim to analyze in addition to reinforcement paradigms. Prior studies have discovered physical dance to be cognitively engaging and hypothesized to promote neuroplastic changes or mitigate future degradation of neural pathways [14,17,21,23,35], thus further supporting the demand for simplified movement related tasks for severe disease cases. As longitudinal studies have urged [55], understanding mitigative effects of interventions are best imparted when repeated throughout illness progression. Through the implementation of reinforcement paradigms within the task and engaging areas in which clear deficits are known, there could be potential in promoting greater response or mitigation in ND groups, such as those with PD.

Within the present study there were several limitations observed. The small sample size of ten participants, while significant, could be increased to obtain a broader scope of the learning curve and how findings may differ. In addition, a participant expressed an issue with the repetitive nature of the task causing dryness in the eyes, which is to be made note of for future studies involved with eye movement tracking. To understand the full extent of our findings would require neuroimaging (fMRI, EEG) analysis of dynamics across regions of interest throughout learning involved in MSL, in addition to other pathways exemplifying neuroplastic changes. EEG is another measurement tool that could be considered in motor learning applications [56]. Extra sessions or an extended follow-up session period up to 1 month would be beneficial in a future eye task study to ensure MSL consolidation and potentially examine loss of memory. A lack of implicit learning is noted, as the explicit learning of the repeated sequence was the central focus of our study. To properly measure implicit learning, it is advised to follow learning of the eye dance sequence with a novel sequence. The measurement of saccades is necessary to include for implicit learning [1,9,57] to assess whether saccadic movements after a novel or random sequence is greater in post-sequence training. Another limitation noted within the present study is the lack of the social aspect of dance toward this paradigm. Crucial to the design of prior studies, the social component of dance may account for differences in neuroplastic changes as well [21,22,34,36,49]. Most importantly, during our learning task, there should have been interactions within the oculomotor system’s cerebellum circuitry and reductions in anxiety, as recently proposed by Zhang et al. [58], which will be examined in our future work.

## 5. Conclusions

These findings suggest that this novel eye dance task is feasible and sufficient for participants learning a specific sequence; therefore, it can be further investigated in several ways. The simplicity and low physical strain on participants are key components of the eye-tracking dance task design and should prove to be cost-effective in training and execution. As a task with the novel removal of dancing within a group, further examination of actions of the dance alone could provide greater understanding of passive and active exercise effect. A psychomotor vigilance task, used in the study of passive and active exercise effects on mental fatigue from Jeyarajan et al. [6], is a task that can be modified under the context of the novel eye-tracking task results. Its design uses randomized targets appearing on a screen and gauges reaction time measurement by clicking targets, which can be replaced with eye movements and blinks to supplement the lack of limb use for future studies. Through the application of our eye-tracking test, a future study involving this task could account for those with disability or limited limb movement and could examine neuroplastic effects. Additionally, this would allow for a comparison of other learning paradigms and tasks within the relevant literature to reinforce similarities in sequence learning and elaborate differences regarding the novel task. In doing so with healthy participants first, a clear definition of how such connections develop and adapt over time can be used in comparison with populations of those with ND.

## Data Availability

The datasets generated and analyzed in the current study will be made available by the corresponding author upon request.

## Abbreviations

The following abbreviations are used in this manuscript:

PD	Parkinson’s Disease
ND	Neurodegenerative Disease
MSL	Motor Sequence Learning
SC	Superior Colliculus
